# A pipeline for automated annotation of yeast genome sequences by a conserved-synteny approach

**DOI:** 10.1186/1471-2105-13-237

**Published:** 2012-09-17

**Authors:** Estelle Proux-Wéra, David Armisén, Kevin P Byrne, Kenneth H Wolfe

**Affiliations:** 1 Smurfit Institute of Genetics, Trinity College Dublin, Dublin 2, Ireland

**Keywords:** Annotation, Saccharomyces, Comparative genomics

## Abstract

**Background:**

Yeasts are a model system for exploring eukaryotic genome evolution. Next-generation sequencing technologies are poised to vastly increase the number of yeast genome sequences, both from resequencing projects (population studies) and from *de novo* sequencing projects (new species). However, the annotation of genomes presents a major bottleneck for *de novo* projects, because it still relies on a process that is largely manual.

**Results:**

Here we present the Yeast Genome Annotation Pipeline (YGAP), an automated system designed specifically for new yeast genome sequences lacking transcriptome data. YGAP does automatic *de novo* annotation, exploiting homology and synteny information from other yeast species stored in the Yeast Gene Order Browser (YGOB) database. The basic premises underlying YGAP's approach are that data from other species already tells us what genes we should expect to find in any particular genomic region and that we should also expect that orthologous genes are likely to have similar intron/exon structures. Additionally, it is able to detect probable frameshift sequencing errors and can propose corrections for them. YGAP searches intelligently for introns, and detects tRNA genes and Ty-like elements.

**Conclusions:**

In tests on *Saccharomyces cerevisiae* and on the genomes of *Naumovozyma castellii* and *Tetrapisispora blattae* newly sequenced with Roche-454 technology, YGAP outperformed another popular annotation program (AUGUSTUS). For *S. cerevisiae* and *N. castellii*, 91-93% of YGAP's predicted gene structures were identical to those in previous manually curated gene sets. YGAP has been implemented as a webserver with a user-friendly interface at http://wolfe.gen.tcd.ie/annotation.

## Background

More genomes have been sequenced from ascomycete yeast species (subphylum Saccharomycotina) than from any other group of eukaryotes. Yeasts provide an excellent system for exploring eukaryotic genome evolution by comparative genomics because their genomes are compact (9–20 Mb with 4700–6500 genes) with few introns, making them straightforward to sequence, but they still retain extensive synteny across deep phylogenetic distances [1-5]. Moreover, there is a wealth of information about gene functions in *Saccharomyces cerevisiae*, probably the most extensively-studied model organism in the world [6].

Yeast comparative genomics has produced many insights into genome evolution, including the discovery of whole-genome duplication (WGD) [7]; development of methods for identifying conserved regulatory elements and RNA genes [8-10]; exploration of changes in the genetic code [11]; and detection of horizontal gene transfer and its functional consequences [12,13]. Furthermore, comparative genomics has played a major role in gene discovery and improving the quality of genome annotations. For example, a comparative analysis of four closely related *Saccharomyces* species [9] led to a revision of the previous annotation of the *S. cerevisiae* genome: elimination of previously annotated ORFs, redefinition of start and stop codons, and discovery of new introns. A similar approach was conducted with the pathogenic basidiomycete yeast *Cryptococcus neoformans*, responsible for cryptococcal meningitis [14].

The need for automated annotation has become urgent with the development of next-generation sequencing technologies, but annotating genomes remains a challenge and still relies on a process that includes many manual steps [15,16]. Annotation can be viewed as consisting of two primary steps: inferring gene structures, and making decisions about the orthology or paralogy relationships between these genes and genes in other species. Yeast genomes present an unusual set of circumstances at both of these steps. The first step, inferring gene structures, is very simple for most yeast genes because they are intronless. However, accurate identification of the coordinates of the other ~4% of yeast genes that have introns is difficult unless cDNA information is available, particularly because many yeast introns are very close to the gene's start codon [17,18]. Some previous automated approaches to annotation of yeast genomes either ignored all introns [19], or used generic fungal gene models that resulted in the over-prediction of hundreds of nonexistent introns [20]. The second step, classifying genes as orthologs or paralogs of genes in other species, is often ignored by automated approaches. They typically use BLAST [21] to identify unidirectional or bidirectional best hits between genes in the new genome and a reference database, and then annotate genes as 'similar to' genes in other species, or as members of particular gene families, without an explicit statement about whether the authors consider the interspecies relationship to be an orthologous one. Decisions about orthology versus paralogy are important because, in general, orthologs tend to have conserved gene function whereas paralogs often diverge [22]. For this reason, manual annotators and scientists working on specific genes usually want to identify orthologs between species, and these orthology decisions frequently make use of synteny information. In yeasts of the family Saccharomycetaceae, orthology relationships are complicated by a WGD event in the common ancestor of several species, leading to a 2:1 synteny relationship between genomic regions in post-WGD and non-WGD species [23,24]. Among all the automatic annotation tools currently available, only a few use synteny data [25,26] and none consider WGD. Until recently, none had been developed specifically for yeast species [27].

In 2005 our laboratory developed the Yeast Gene Order Browser (YGOB), which is a database and interface for comparative genomics for Saccharomycetaceae yeasts [28,29]. A major strength of YGOB is that it contains manually curated sets of orthologs (and WGD-derived paralogs in species that underwent WGD), which have been identified based on their conserved synteny relationships. We recently carried out a project to sequence the genomes of multiple previously unstudied yeast species by Roche-454 sequencing and *de novo* assembly [30]. We reasoned that the information in YGOB could be used to automatically annotate the new yeast genomes with accuracy comparable to a manual annotation. The core concept of our approach is that any particular region of a newly yeast sequenced genome is likely to contain genes whose gene order is similar to that in other yeast species, and therefore can be mapped onto the 'Ancestral' gene order that we previously inferred to have existed just before the WGD occurred [31]. After the approximate correspondence between a region of the newly-sequenced genome and a region of the Ancestral genome has been established, the gene content of that Ancestral region can then be used to improve the annotation of the corresponding region in the new genome – for example to make decisions about the correct orthology relationships for genes that are members of multigene families, or to find genes that were not initially annotated but which are expected to be present in the region because they are present in the syntenic region in other species [32].

In this manuscript we present YGAP (Yeast Genome Annotation Pipeline), the pipeline we developed to carry out automated annotation by this approach. The data input to YGAP are the entire YGOB database, the scaffold sequences from the newly sequenced species, and (if available) its contigs and individual sequence reads. The output includes a set of annotation files, both in YGOB's internal format and as standard EMBL database format. The webserver also provides a 'mini-YGOB' interface for the new genome that allows its gene order to be compared to other species. To test the pipeline we used the genome of the extensively studied *S. cerevisiae* as well two of the new genomes from our sequencing project, *Naumovozyma castellii* and *Tetrapisispora blattae*[30].

## Methods

### Input data

Next-generation sequencing projects can produce three different types of output DNA sequence files: (i) a 'reads' file containing all the primary sequence reads; and after assembly, (ii) a 'contigs' file containing the contigs assembled from overlapping reads; and (iii) a 'scaffolds' file, typically made by concatenating together those contigs whose relative order and orientation is known, separated by runs of 'N' bases representing the estimated lengths of unsequenced gaps. For example, our genome project for *Naumovozyma castellii* generated 1.4 million Roche-454 reads. The Celera assembler [33] assembled these reads into 3851 contigs, and arranged 43 of these contigs into 9 scaffolds that correspond to almost complete chromosomes (the other 3808 contigs were not incorporated into scaffolds; none of them is larger than 2.1 kb).

To run YGAP, the user must provide a scaffolds file from the new species. This is the only sequence file whose input is mandatory, but if contigs and reads files are also available YGAP can use them for optional steps. The user must also specify whether the new genome comes from a post-WGD or a non-WGD species, which can usually be predicted from the species' phylogenetic position.

YGAP also requires access to the YGOB database. This database consists of previously annotated yeast genome sequences, and lists of the gene names that comprise each of its ~9500 homology pillars [28]. A pillar consists of a manually curated set of genes that are orthologs, or paralogs resulting from WGD, among the species in the database. Prior to our sequencing project [30], YGOB contained data from 11 species: *S. cerevisiae*[34], *S. bayanus*[9,35], *Naumovozyma castellii* (formerly called *Saccharomyces castellii*) [35,36], *Vanderwaltozyma polyspora* (formerly called *Kluyveromyces polysporus*) [37], *Candida glabrata*[38], *Zygosaccharomyces rouxii*[16], *Kluyveromyces lactis*[38], *Eremothecium gossypii* (previously called *Ashbya gossypii*) [24], *Lachancea kluyveri* (previously called *Saccharomyces kluyveri*) [16], *Lachancea thermotolerans*[16], and *Lachancea waltii* (previously called *Kluyveromyces waltii*) [23].

In the tests of YGAP described here, to avoid circular reasoning we omitted *S. cerevisiae* data from YGOB pillars when annotating the *S. cerevisiae* genome, and we omitted *N. castellii* data (from the draft sequence of the genome [35]) when annotating our new sequence of the *N. castellii* genome.

### Checking the integrity of scaffolds

YGAP will report on the consistency between the primary data and the scaffold structure, if the user provides a reads file that includes 'paired' reads that are expected to be close together in the genome. The user must also provide a contigs file for this step. Using BLASTN, YGAP maps each read from a pair onto a contig, provided that the read has a unique hit in the genome (scaffolds file). Pairs of reads that map to different contigs identify possible physical links between contigs, which should correspond to the scaffold organization deduced by the assembly program. YGAP summarizes these data in the form of a matrix listing the number of read-pairs that support a connection between any two contigs. It sorts the contigs in the order that they occur in the scaffolds. This analysis allows the user to see the amount of support for any connection between two contigs in the scaffold structure, and the support for possible alternatives.

### Locating genes

In an initial annotation step, tRNAscan-SE [39] is used to detect (with default parameters) and annotate tRNA genes. In the later steps of YGAP's annotation process, no protein-coding gene will be allowed to overlap with a tRNA gene.

Annotation of protein-coding genes is largely based on TBLASTN searches [21]. We use every protein from every YGOB pillar as a query in a TBLASTN search against the genome (scaffolds file), and initially store all hits with Expect values E < 1e-05. For each pillar, we then identify the place in the genome where that pillar has its strongest hit, and other places where it has weaker hits. That is, among the proteins encoded by the pillar (*P*) we identify the query protein (*Q*) that gives the lowest TBLASTN E-value (*E*) versus the genome and store the location of that hit. It is likely that query *Q* comes from the species that is most closely related to the new genome. We also store the location of weaker hits between *Q* and the genome, provided that the exponent of their E-values is lower than −30 and lower than *E*/2 (that is, if the strongest hit's E-value was 1e-100 we would retain other hits with E-values < 1e-50). For each stored location for a hit by query *Q*, we build a gene model as described later below.

Several YGOB pillars can match the same location in the new genome due to the existence of paralogous genes. Thus two (or more) pillars might hit identical or overlapping regions of the genome, and gene models would initially be constructed for both of them. After the TBLASTN searches are complete, we use synteny information to determine which pillar is the correct match for this genomic location. Specifically, if locus *L* in the new genome is hit by queries from two pillars *P1* and *P2*, we examine the regions of the new genome upstream and downstream of *L* and identify neighboring pillars (*Pleft* and *Pright*) that have been mapped unambiguously to these flanking regions. We then ask whether, in the Ancestral genome [31] or in *S. cerevisiae*, *P1* or *P2* occurs in the interval between *Pleft* and *Pright*; if this is true for *P1* but not *P2*, we assign locus *L* to pillar *P1* and discard *P2* as a candidate for *L*. Note that this assignment is based on synteny, without regard to the TBLASTN E-values for *P1* and *P2*.

When a post-WGD species is being analyzed, the software allows two candidate genomic loci to be assigned to a single pillar, whereas in a non-WGD species only one locus can be assigned to a pillar. Where a tandem duplication has occurred, only one of the duplicates is assigned to the pillar while the other is assigned to a separate pillar containing the second copy in any species in which the tandem duplication has occurred. If a locus is hit by several pillars, but none of them has conserved synteny, the location is annotated as a gene but is not assigned to any existing pillar.

### Gene models

For each stored hit between a query protein *Q* from a pillar *P* and a genomic location *L*, we make a gene structure prediction, based on YGOB's information about intron/exon structures in pillar *P*. We choose a reference gene *R* from pillar *P* to use as a basis for the model, giving preference where possible to genes from the same species group (post-WGD or non-WGD) as the new genome. We also give preference to intron-containing gene models over gene models without introns, because we want to consider the possibility that the new gene might contain an intron if any of the existing annotated genes in the same pillar and species group contains an intron. For example, if the new genome comes from a post-WGD species, and some of the genes from post-WGD species in pillar *P* contain an intron, *R* is chosen to be the post-WGD intron-containing gene with the best TBLASTN E-value. If no such gene exists, the order of preference in choosing *R* from the set of genes in *P* is as follows: (*i*) the post-WGD gene with the best hit; (*ii*) the intron-containing gene with the best hit; (*iii*) the gene with the best hit. If more than one gene has the same BLAST E-value (such as 0.0), the gene with the best score is used. The mean lengths of the non-WGD genes and post-WGD genes in *P* are also calculated at this step. Once a reference gene *R* has been defined for locus *L*, we store the coordinates of the best TBLASTN hit between *R* and locus *L* regardless of its E-value.

**Figure 1:**
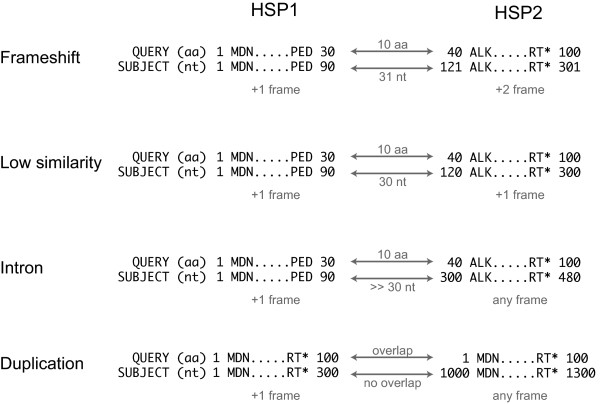
**Classifying pairs of consecutive HSPs.** In this hypothetical example, a TBLASTN search using a 100 amino acid query protein produces two HSPs that are close together in the genome. We classify the relationship between two consecutive HSPs into one of four categories: (i) Frameshift. Two consecutive HSPs are in different frames but the distance between them is similar in both the query and the subject. (ii) Region of low similarity. Two consecutive HSPs are in the same frame, separated by a similar distance in both the query and the subject, with no stop codon between them. (iii) Intron. Two consecutive HSPs for which query and subject coordinates are dissimilar. This possibility is only considered if an existing gene from the same pillar and species group contains an intron. (iv) Duplication. If all other possibilities have been excluded, two consecutive HSPs suggest a probable local gene duplication.

The next step in creating a gene model at locus *L* depends on the number of BLAST HSPs (high scoring pairs) generated between the reference gene *R* and the genome in the vicinity of locus *L*. If only one HSP is present, the endpoints of this HSP are used directly as seed coordinates to build a gene model. If two or more HSPs are detected on the same strand, we evaluate them in pairs in order of location (HSP1 with HSP2; HSP2 with HSP3, etc., along the chromosome at locus *L*). We classify the relationship between each pair of consecutive HSPs as one of the following four situations (Fig. 1): (*i*) frameshift (the two HSPs are the result of a probable frameshift sequencing error); (*ii*) low similarity (the two HSPs and the region between them are all part of the same gene and can be merged without requiring a frameshift); (*iii*) intron (the two HSPs correspond to matches between individual exons and the genome sequence); (*iv*) gene duplication (in all other instances of consecutive HSPs, we assume that the HSPs correspond to separate genes that were formed by full or partial gene duplications). In situations *i-iii*, the outer edges of the two HSPs are used as seeds for the gene model; in situation *iv*, two separate models are created.

**Figure 2:**
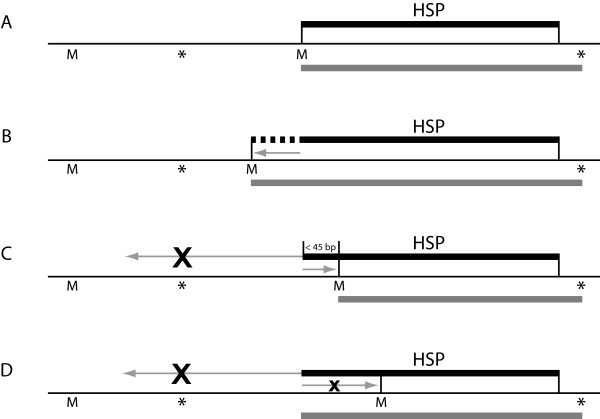
**Method for defining start and stop codon coordinates.** The thick black bar indicates the location of the original BLAST HSP, and the thick grey bar indicates the gene coordinates reported by YGAP. M and asterisk (*) represent the locations of all possible start (ATG) and stop (TAA/TAG/TGA) codons in the same frame as the HSP. The start codon is chosen by searching around the beginning of the HSP as follows: (**A**) If the HSP (or the upstream HSP, in the case where a pair of HSPs is being considered) begins with a methionine codon, no change is made to the starting coordinate. (**B**) If the HSP does not begin with methionine, the ORF is extended to the furthest upstream methionine. (**C**) If during extension a stop codon is encountered before reaching a methionine, the software instead searches for a leading methionine within the first 45 nucleotides of the HSP. (**D**) If no suitable starting methionine is found using these steps, the original coordinates of the HSP are kept and the gene is tagged for manual inspection. Stop codons are found by walking downstream from the HSP, unless there is a stop codon within the HSP (in which case the HSP is trimmed accordingly).

When these possibilities have been evaluated, the seed coordinates derived from the HSPs are then used to construct a gene model. We attempt to extend the seed coordinates upstream and downstream to find start and stop codons for the gene (Fig. 2), by matching the HSP's location and frame to a map of all open reading frames in the genome (generated using the GetORF program from the EMBOSS package [40]). If we fail to find a suitable start position by elongating the HSP in this way, we instead look for the requisite codon within the HSP. For instance, if no possible start codon is found upstream of the HSP, we will trim the 5' end of the HSP by up to 45 nucleotides in order to find a suitable start codon. In the event that a suitable start codon is still lacking after this step, the gene is instead annotated with the seed coordinates and is tagged for manual curation (indicating that the automated process could not construct a satisfactory gene model at this locus). 

### Intron annotation

Because introns in yeast genes are rare, we only consider the possibility that a gene model may require an intron if another gene pillar *P* already contains an annotated intron. This other gene must come from the same species group (that is, post-WGD or non-WGD) as the new genome; the user specifies whether the new genome is post-WGD or non-WGD when launching YGAP. We use the HSPs generated in the TBLASTN search to test for the existence and location of the intron. If there are two HSPs, we search for a possible 5’ splice site (GTATGT, GTCAGT, GTTCGT, GTACGT, GTAAGT, GCATGT, GTATGA, GTATGC), branchpoint site (ACTAAC, GCTAAC, ATTAAC) and 3’ splice site (CAG, TAG) [17]. In many cases, however, the first exon of a gene is too small to generate a TBLASTN hit so there is only one HSP, corresponding to exon 2. In these cases we attempt to identify a suitable intron and a suitable start codon, in order to make a protein similar to that of other species. As almost all intron-containing genes in YGOB have only one intron (2099 out of 2176 genes, totaled over all species), we search only for one intron per gene. Restricting the search to one intron per gene greatly simplifies the process, as without HSP data the number of combinations of exon features that could be generated to make a feasible multi-intron gene is too large to make an accurate prediction. In practice, this limitation means that any 2-intron genes in the genome will be flagged by YGAP as requiring manual intervention.

### Frameshift correction

If the analysis of HSP pairs detects an apparent frameshift sequencing error (Fig. 1) in the scaffold data, YGAP can try to correct the error automatically. This option can be enabled or disabled by the user when YGAP is launched. If enabled, the output from YGAP will include a modified version of the scaffolds file in which bases have been added, or more rarely removed, at particular sites in order to correct frameshifts. YGAP's output also includes lists of the genes in which frameshifts have been automatically corrected, and lists of genes in which a probable frameshift was detected but no automated correction was possible. The user can also choose to disable automatic frameshift correction, but still generate a list of genes in which probable frameshift errors have been detected.

The presence of a frameshift error usually results in two HSPs in different frames. YGAP provides the option of making two types of automatic correction: (*i*) If a file of primary sequence reads from the same species was provided as part of the input, we carry out a BLASTN search against the reads file using as a query the region from the scaffolds file corresponding to 50 bp upstream of the end of the first HSP to 50 bp downstream of the start of the second HSP. For BLASTN hits with E < 1e-30, we examine the match between the query and the read, noting the indels (insertions/deletions). For each indel seen in the search, we count how many reads contain it. We take the most common indel and test whether it would result in creation of an intact ORF. If it does, we make the corresponding change in the scaffold sequence and the frameshift has been fixed. If not, we do not make the change and instead we examine the next-most common indel. We continue this process until the frameshift has been fixed or until there are no more indels that were seen in at least 2 reads. (*ii*) If no reads file is available, one or two N nucleotides are inserted into the gene containing a frameshift, at the estimated site, in order to fix it approximately.

### SearchDOGS and large ORF steps

Two final steps of searching for protein-coding genes are carried out after the initial annotation has been completed. First, we run a version of SearchDOGS [32] to look for small, highly-divergent genes that can be recognized based on their conserved synteny to orthologs in other species. SearchDOGS does not employ any threshold for BLAST similarities, so it can find weak hits that were missed by the TBLASTN method described above (which used a cutoff of E < 1e-5), provided that they are in a conserved genomic location and do not contain introns. Second, we use GetORF [40] to identify any large ORFs (≥150 amino acids, not overlapping with any other feature) that remain unannotated in the genome. Genes predicted by SearchDOGS and GetORF are included in the genome annotation and also listed separately in the YGAP output to allow them to be examined manually.

### Retrotransposons

Retrotransposons (primarily Ty elements and similar elements in other species [41]) pose a particular challenge to automated annotation because: (*i*) They are mobile, so their locations are usually not conserved across species or even among different strains of the same species; (*ii*) They occur in multiple copies in most species, with copies in different places in the genome often being highly similar in sequence. Tandem arrays of integrated retrotransposons are also common, as are solo LTR (long terminal repeat) units; (*iii*) Their repetitive nature tends to cause problems to sequence assembly software, so they often occur at the ends of contigs and their sequences are often incomplete; (*iv*) Some copies of these elements are inactive and their protein-coding regions are pseudogenes. In YGOB, annotated protein-coding genes that are parts of retrotransposons are flagged with a special 'Ty' label and displayed in a dark gray color. YGOB always leaves these genes as singletons and does not put them into pillars with genes from other species [29].

During protein-coding gene annotation in YGAP, if a YGOB gene carrying the 'Ty' label hits a region of the new genome (with TBLASTN E < 1e-5), that region will be flagged as Ty-like. YGAP will attempt to identify coordinates for the gene as described above, but the gene will be flagged as 'Ty' and will be left as a singleton. YGAP does not attempt to work out the detailed structure of retrotransposons.

### Mini-YGOB browser

The output from YGAP is presented in an individual webpage with links to a set of files including the full annotation file (listing all the genes), files of genes tagged for manual attention (one list per tag, such as genes tagged as having possible uncorrected frameshifts), and other files depending on what options the user has selected. Additionally, we provide the user with a private browser interface to inspect the new genome. This browser is a simplified version of YGOB, in which the only species displayed are the new genome, *S. cerevisiae* (as a post-WGD reference species), *E. gossypii* (as a non-WGD reference), and the inferred Ancestral genome [31]. This 'mini-YGOB' view allows the user to examine the structure of the annotated genome in the region around any gene of interest, for example to examine the context of singleton genes, and it provides an easy way to retrieve the predicted protein sequence of any gene of interest. The mini-YGOB interface also helps the user judge the quality of the genome assembly and annotation by showing the extent of colinearity between the new genome and the reference genomes, and by allowing the user to see the extent to which genes conserved among other species are missing (or remain unannotated) from the new genome.

We anticipate that a user might run YGAP to produce an initial annotation of the genome, then use the mini-YGOB interface to look for structural problems in the assembly (such as telomere-to-telomere fusion artifacts, or to identify where contigs that were not incorporated into scaffolds may fit into the genome), and then perhaps modify the scaffolds file and re-run YGAP. The mini-YGOB browser is only accessible to the user, and only for a period of 6 months after YGAP is run, because we intend it to be used for initial inspection of the genome and annotation, rather than as a permanent database for the genome. New genomes can be added into YGOB proper once they are finalized.

## Results

**Figure 3:**
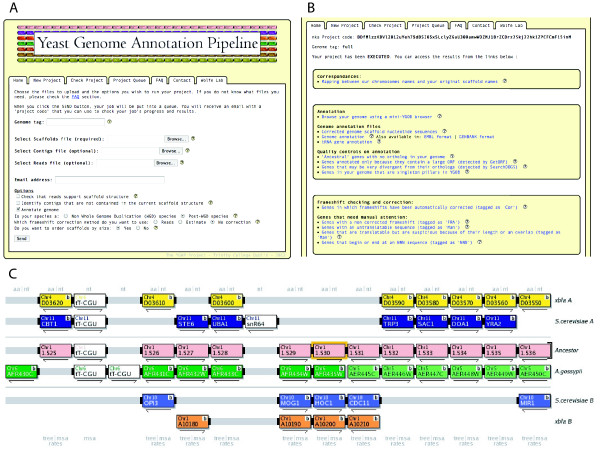
**Screenshots from the YGAP website.** (**A**) Upload screen. (**B**) Results page including links to several types of output files and gene lists. (**C**) Mini-YGOB browser showing the new annotated species (here, *T. blattae*, a post-WGD species, in yellow/orange), compared to genomes of *E. gossypii* (non-WGD species, in green), *S. cerevisiae* (post-WGD species, in blue), and the Ancestral genome (in pink).

YGAP was designed to have a simple interface to our webserver. An upload screen (Fig. 3A) allows the user to upload the sequence files (scaffolds, and possibly reads and contigs), to provide a prefix that will be used for gene names, to designate the species as post-WGD or non-WGD, and to select options related to automated frameshift correction. After the run is complete, the user receives an email directing them to a results page (Fig. 3B). A sample results page can be viewed at http://wolfe.gen.tcd.ie/annotation/example.html. The results page includes links to several types of output files and gene lists, as well as a link to a “mini-YGOB” browser (Fig. 3C).

### Tests with S. cerevisiae

To test YGAP's performance we ran an automatic annotation of the genome of *S. cerevisiae*, which is very well studied and annotated. To do this, we retrieved the chromosomal DNA sequences of same version of the *S. cerevisiae* genome that is currently used in YGOB (strain S288c; based on sequence and annotation from the SGD database, excluding genes annotated as 'dubious'). To avoid using the annotated *S. cerevisiae* gene set as a source of information, we removed all *S. cerevisiae* genes from the YGOB pillar set for this experiment. We then ran YGAP using the *S. cerevisiae* genome DNA as input, and with the frameshift correction option disabled. This setup replicates the simplest scenario that the pipeline may encounter, where no file of primary sequence reads is available but the scaffolds are expected to be highly accurate.

The whole annotation took approximately 5 hours on a 3 GHz processor with access to 4 GB of RAM and was then compared with the curated *S. cerevisiae* annotation in YGOB. For comparison, we did a similar annotation using AUGUSTUS [42] with default options set. AUGUSTUS is a widely used automatic annotation tool that has been specifically trained for *S. cerevisiae*. YGAP predicted 5659 potential genes in *S. cerevisiae*, compared with 5551 predicted by AUGUSTUS and 5604 curated genes in YGOB (including genes from Ty elements). To assess the accuracy of the gene models, we compared the predicted gene coordinates to those in the YGOB database (Table 1). The analysis of coordinates (start codon position, stop codon position, and the coordinates of introns where present) shows that our pipeline predicted the structures of 5119 genes completely correctly, which is 181 more than AUGUSTUS and 91% of the genes in YGOB. However if we consider only start and stop codon positions and ignore intron structures, the gap between the YGAP and AUGUSTUS predictions is reduced to about 70 genes. 

**Table 1:** **Comparison of automatic reannotations of the *Saccharomyces cerevisiae *genome by YGAP and AUGUSTUS, to the reference annotation**

**Chromosome**	**Same Coordinates**		**False Negatives**		**Overlap**		**False Positives**		**Wrong Start or Stop coordinate**	
	**YGAP**	**AUGUSTUS**	**YGAP**	**AUGUSTUS**	**YGAP**	**AUGUSTUS**	**YGAP**	**AUGUSTUS**	**YGAP**	**AUGUSTUS**
Chr_1	75	76	7	5	1	1	2	3	8	9
Chr_2	354	346	3	13	2	2	5	6	28	26
Chr_3	135	126	1	9	6	0	6	4	9	16
Chr_4	668	646	8	24	2	1	15	16	47	54
Chr_5	236	232	1	6	7	0	6	10	18	24
Chr_6	101	106	2	8	1	1	8	5	14	3
Chr_7	457	446	1	9	6	1	7	14	37	45
Chr_8	237	235	2	7	2	1	9	8	22	20
Chr_9	180	176	0	7	5	1	4	4	20	21
Chr_10	312	300	0	9	4	0	6	5	25	32
Chr_11	284	275	1	9	5	0	1	0	18	24
Chr_12	445	414	3	14	5	2	10	10	24	48
Chr_13	405	387	2	10	7	0	5	9	22	39
Chr_14	353	337	0	11	4	3	3	4	25	31
Chr_15	465	451	3	14	4	0	5	12	34	42
Chr_16	412	385	10	17	4	0	7	7	25	49
Total	5119	4938	44	172	65	13	99	117	376	483

Columns show the numbers of genes in each category, when compared to genes in the reference YGOB annotation of the *S. cerevisiae* genome (which is based on *Saccharomyces* Genome Database annotation).

The numbers of false negative and false positive gene predictions by YGAP also compare favorably to AUGUSTUS. The numbers of false negatives (situations where the automated method fails to predict any gene model in a region of the genome where YGOB shows an annotated *S. cerevisiae* gene) for YGAP was four times lower than for AUGUSTUS (44 versus 172; Table 1). A second category of false negatives consists of “overlap” cases, where the automated programs failed to annotate a gene and instead extended a neighboring gene (usually by incorrect start codon assignment) so that it overlapped with the range of the unannotated gene. YGAP overlooked 65 genes for this reason, compared to 13 for AUGUSTUS. Combined, these two false negative categories amount to 109 genes for YGAP and 185 for AUGUSTUS. The numbers of false positives (gene models predicted in regions of the genome where no gene is present in the YGOB annotation of *S. cerevisiae*) were more similar: 99 for YGAP and 117 for AUGUSTUS. Of the remaining gene predictions whose structures were incorrect, most had either a wrong start or stop coordinate (376 from YGAP and 483 from AUGUSTUS).

We also compared the performance of YGAP and AUGUSTUS in predicting the intron/exon structures of genes in *S. cerevisiae* (Table 2). In the *S. cerevisiae* genome there are 265 introns in the protein-coding regions of 256 genes [6]. YGAP predicted a total of 146 introns, of which 2 were false positives (the gene actually has no intron). The main problem was that YGAP's false negative rate (122 true introns not predicted) is high. Of the 144 true-positive predictions from YGAP, the predicted intron coordinates were completely correct for 127 (87% of the predictions, or 47% of all introns studied). AUGUSTUS had a similarly high false negative rate, and predicted more introns in total due to a higher false positive rate (Table 2). 

**Table 2:** **Comparison of intron structure predictions in *S. cerevisiae *and *N. castellii *by YGAP and AUGUSTUS **

**Species**	**Software**	**Predicted introns (a+b+c)**	**False positives (a)**	**Completely correct (b)**	**Real intron, incorrect coordinates (c)**	**False negatives (d)**	**Total introns studied (b+c+d)**
*S. cerevisiae*	YGAP	146	2	127	17	122	266
	AUGUSTUS	221	90	87	44	121	252
*N. castellii*	YGAP	146	12	123	11	58	192
	AUGUSTUS	251	173	58	20	94	172

Note that False Negatives in YGAP include not only those genes for which no intron was predicted by the software, but also those for which intron coordinates could not be defined and were tagged for manual curation. The total number of introns studied (rightmost column) differs between YGAP and AUGUSTUS because some genes were not predicted by both methods.

### Automatic annotations of Naumovozyma castellii and Tetrapisispora blattae

We used YGAP to automatically annotate the genome of *Naumovozyma castellii*. This species has previously been called *Saccharomyces castellii* and *Naumovia castellii*. Its genome was originally sequenced, by Sanger sequencing to draft (3x) coverage by Cliften et al. (2003) which resulted in hundreds of contigs. Genes in these contigs were annotated manually by our laboratory as part of the YGOB project [28], and we later added 18 genes using SearchDOGS [32]. We refer to this annotation as the 'Scas' annotation (corresponding to the prefix of the gene names as annotated in YGOB). The total number of protein-coding genes in the Scas dataset is 5691.

We recently resequenced the same strain of *N. castellii* using the Roche-454 platform with 20x coverage, with a strategy designed to maximize the size of scaffolds. This genome was assembled without making use of the Sanger data. The resequenced genome comprises only 10 scaffolds, which compares reasonably well to a pulsed-field gel electrophoresis estimate that this species has 9 chromosomes [43]. We then used these *N. castellii* scaffolds as input to YGAP. As before, to avoid circular reasoning we ignored annotated *N. castellii* ('Scas') genes from the input YGOB pillars, but we included *S. cerevisiae* genes. For this run of YGAP, we included the sequence reads file and allowed automatic correction of frameshifts. The whole annotation took 6.25 hours on the same server, and 5682 protein-coding genes (including 19 from retrotransposons) were predicted (Table 3). We refer to this dataset as the 'Ncas' dataset (again corresponding to the prefix of the gene names). In this run, YGAP identified 184 positions in the genome as frameshift sites, and it automatically corrected 109 of these (81 nucleotide additions and 28 nucleotide deletions); it flagged the remaining 75 sites as probable frameshift sites that it was unable to correct. 

**Table 3:** **Comparison of automatic annotations of the *Naumovozyma castellii *genome by YGAP and AUGUSTUS, to the reference (Scas) annotation **

**Scaffold**	**Same Coordinates**		**False Negatives**		**Overlap**		**False Positives**		**Wrong Start or Stop coordinate**	
	**YGAP**	**AUGUSTUS**	**YGAP**	**AUGUSTUS**	**YGAP**	**AUGUSTUS**	**YGAP**	**AUGUSTUS**	**YGAP**	**AUGUSTUS**
scf7180000013410 (chr. 1)	1427	1289	12	34	31	5	19	46	56	198
scf7180000013411 (chr. 2)	851	764	8	28	12	5	13	19	35	106
scf7180000013405 (chr. 3)	544	485	3	17	13	2	14	25	28	84
scf7180000013408 (chr. 4)	462	418	1	10	9	1	3	9	16	59
scf7180000013414 (chr. 5)	386	338	3	16	13	8	6	18	11	50
scf7180000013415b (chr. 6)	374	336	3	7	6	1	10	15	17	56
scf7180000013412 (chr. 7)	387	342	3	16	8	1	11	25	18	57
scf7180000013409 (chr. 8)	331	294	1	8	11	11	6	9	15	44
scf7180000013415a (chr. 9)	290	250	4	12	1	3	3	8	17	47
scf7180000013407 (chr. 10)	208	185	2	5	5	1	5	8	10	34
Total	5260	4701	40	153	109	38	90	182	223	735

Columns show the numbers of genes in each category, when compared to genes in the reference (Scas) manual annotation of *N. castellii* genes.

**Table 4:** **Numbers of annotated genes requiring frameshift corrections or manual attention in *S. cerevisiae *and *N. castellii***

	***S. cerevisiae***		***N. castellii***	
	**YGAP output**	**Confirmed**^**a**^	**YGAP output**	**Confirmed**
Automatically corrected^b^	-	-	97	86
Unable to correct^c^	93	3	76	33
Tagged for manual inspection^d^	390	155	465	216

^a^ Confirmed by comparison to the curated annotations of *S. cerevisiae* and *N. castellii*.

^b^ Frameshifts corrected using the reads file.

^c^ Either because the reads were not helpful or there were no frameshift (e.g. genes in which natural ribosomal frameshifting occurs).

^d^ These potential genes may contain undetected introns, untranslatable sequences due to inaccurate prediction of exon locations, or may begin or end in undefined (N) nucleotides.

Comparing the annotations shows that YGAP's predictions were identical to the manually predicted gene structures for 5260 genes (93% of the predicted genes), with 90 false positives and 40 false negatives (Table 3), while AUGUSTUS gets right 4701 genes (83%), with 182 false positives and 153 false negatives. As with the *S. cerevisiae* annotation, the numbers of false positives, false negatives and incorrect start/stop codons from YGAP were consistently lower than from AUGUSTUS. YGAP also outperformed in the prediction of intron coordinates, predicting far fewer false positive introns and getting the coordinates completely correct more often (Table 2).

We also used YGAP to annotate the genome of *Tetrapisispora blattae*, a post-WGD species for which no previous genomic data existed so there is no reference annotation to which we can directly compare YGAP's results. As input to YGAP we used the 8 large scaffolds and 373 contigs obtained from the Celera assembler, as well as 319,888 pairs of primary sequence reads. Automated frameshift correction and scaffold integrity checking were enabled in the YGAP run, which took 12.5 hours. The genome integrity checking steps identified two joins in the scaffold data that were not well supported by nonrepetitive paired sequence reads, so in the final version of the genome we broke these joins. The frameshift correction steps inserted a total of 398 nucleotides (including 194 As and 184 Ts) and deleted 48 nucleotides from the 14.1 Mb genome, affecting the structures of 418 genes. YGAP predicted 5600 protein-coding genes in *T. blattae*, corresponding to 4534 loci in the ancestral genome [31]. There are 383 loci in the ancestral genome at which *T. blattae* has no annotated gene, and there are 830 annotated singletons in *T. blattae.*

## Discussion

Our aim was to develop a new bioinformatics pipeline for the automated annotation of yeast genomes, exploiting information from existing genomes to the greatest extent possible. The pipeline has been specifically designed for the *de novo* annotation (not reannotation) of genomes of new yeast species. YGAP is very much a yeast-specific tool, designed to cope with the specific challenges (rare introns, WGD) and opportunities (conservation of gene order over long distances, once WGD is taken into account, even in the presence of high gene sequence divergence) that yeast species present. We recently adapted YGAP to annotate the genome sequence of *Candida orthopsilosis*[44] using other *Candida* clade genomes from the CGOB database [45] as a reference set, but YGAP will not be readily adaptable to more distantly related, intron-rich fungi or other eukaryotes.

The full YGAP pipeline includes not only *de novo* genome annotation but also some error-checking tools that use all the outputs from high-throughput sequencing methods, *i.e.* files of scaffolds, contigs, and reads. These tools include a verification that scaffold structure is well supported by the paired primary sequencing reads, and identification of any large contigs that were not included in the scaffolds. These steps are not essential in order to run the automatic annotation, but they can improve the quality of the sequenced genome and thus the quality of the annotation. YGAP is also unique in its ability to make use of a file of primary sequence reads to try to correct apparent frameshift errors in the assembly. However, these features require the user to upload the primary reads data, and at the moment this is only possible for Roche-454 or Sanger projects (the primary files from Illumina sequencing are simply too large to upload to our server). Nevertheless, we expect that these additional quality-control steps will eventually become unnecessary because they are largely dependent on the quality of the sequence and the assembly, so the need for them should decline as the quality of next-generation sequencing techniques improves. The basic annotation steps in YGAP require only a scaffolds file, which can come from any sequencing platform.

A key feature of YGAP is that it tries to use the orthologous genes from other species, identified by a synteny method, to make gene structure predictions. Some other previous automatic genome annotation tools have also been based on the identification of orthologs. Some of these are quite specific, such as Dogma [46], an annotation tool designed to annotate organellar genomes, and MaGe [25] which annotates microbial genomes. Other automatic genome annotation tools can be more widely used and can annotate both prokaryotic and eukaryotic genomes. RAPYD [27] is an annotation platform that has been developed specifically for yeast species and uses AUGUSTUS [42] as its main tool for gene prediction.

We tested YGAP using the *S. cerevisiae* annotation in YGOB as a 'gold standard' for reference and compared the results to predictions made by AUGUSTUS using its *S. cerevisiae* model. The results showed that YGAP was able to correctly predict more than 90% of *S. cerevisiae* gene structures correctly. Importantly, the use of multi-species annotations in YGOB together with synteny information resulted in a significant reduction in the numbers of both false-positive and false-negative gene predictions. A manual check showed that most spurious YGAP gene annotations (false positives) correspond to annotated pseudogene features. YGAP’s failure to detect certain genes (false negatives) was due to: (i) the genes being species-specific gene gains or species-specific families, (ii) the genes being located in subtelomeric regions where rapid gene family expansion has resulted in multiple gene duplications, and (iii) in post-WGD species, occasional failure to annotate both copies of a gene retained in duplicate after WGD if both copies are located on the same scaffold.

YGAP's performance on intron-containing genes is less impressive, resulting in correct prediction of both the presence of an intron and the locations of its boundaries only about half the time, but it nonetheless outperforms AUGUSTUS and makes few false-positive predictions of introns. One cause of the poor performance on introns may be inaccurate annotation of intron-containing loci in the existing genome annotations in YGOB. It should also be noted that YGAP will only predict a maximum of one intron per gene, whereas in fact a small number of genes (9 in *S. cerevisiae*) are known to have two introns in their coding regions.

## Conclusions

YGAP has been able to correctly annotate 90% and 93% of the genes in *Saccharomyces cerevisiae* and *Naumovozyma castellii* respectively. It is more difficult to quantify YGAP's performance on the other genomes we sequenced *de novo*[30] because there is no other annotation to which its output can be compared. All YGAP's results are provided to the user for manual inspection in different lists of tagged genes. These include a list of genes in which frameshifts have been automatically corrected; a list of those in which a frameshift probably exists but was not automatically corrected; a list of genes whose DNA sequences are not properly translatable (for example, due to the presence of an unannotated intron); and list of genes that extend into regions of scaffolds that contain continuous runs of N nucleotides, making identification of start/stop codons impossible. YGAP proved effective in the annotation of seven new genomes from Saccharomycetaceae species [30] and we anticipate that it will be applicable to many other genomes in the future. Nevertheless, manual refinement of the results remains necessary and we are still some distance from our ultimate goal of being able to turn a sample of genomic DNA from an uncharacterized yeast species into a complete and fully annotated genome sequence without any human intervention.

### Availability

YGAP's webserver is available without restrictions on use at http://wolfe.gen.tcd.ie/annotation.

## Abbreviations

YGAP: Yeast Genome Annotation Pipeline; YGOB: Yeast Gene Order Browser; WGD: Whole Genome Duplication.

## Competing interests

The author declared that they have no competing interest.

## Authors’ contributions

EPW designed and implemented YGAP software with supervision from KHW. DA made the webserver interface. KPB integrated YGAP with YGOB. EPW and KHW wrote the manuscript, which was read and approved by all authors.
